# Dependence of Protein Structure on Environment: FOD Model Applied to Membrane Proteins

**DOI:** 10.3390/membranes12010050

**Published:** 2021-12-30

**Authors:** Irena Roterman, Katarzyna Stapor, Krzysztof Gądek, Tomasz Gubała, Piotr Nowakowski, Piotr Fabian, Leszek Konieczny

**Affiliations:** 1 Department of Bioinformatics and Telemedicine, Medical College, Jagiellonian University, Medyczna 7, 30-688 Krakow, Poland; 2 Department of Applied Informatics, Silesian University of Technology, Akademicka 16, 44-100 Gliwice, Poland; katarzyna.stapor@polsl.pl; 3 Sano Centre for Computation Medicine, Czarnowiejska 36, 30-054 Kraków, Poland; krzysztof.gadek15@gmail.com (K.G.); t.gubala@sanoscience.org (T.G.); p.nowakowski@sanoscience.org (P.N.); 4 Department of Algorithmics and Software, Silesian University of Technology, Akademicka 16, 44-100 Gliwice, Poland; piotr.fabian@polsl.pl; 5 Chair of Medical biochemistry, Medical College, Jagiellonian University, Kopernika 7, 31-034 Krakow, Poland; mbkoniec@cyf-kr.edu.pl

**Keywords:** membrane proteins, hydrophobicity, hydrophobic core, MscS, mechanosensitive channels, efflux in bacteria, MsbA

## Abstract

The natural environment of proteins is the polar aquatic environment and the hydrophobic (amphipathic) environment of the membrane. The fuzzy oil drop model (FOD) used to characterize water-soluble proteins, as well as its modified version FOD-M, enables a mathematical description of the presence and influence of diverse environments on protein structure. The present work characterized the structures of membrane proteins, including those that act as channels, and a water-soluble protein for contrast. The purpose of the analysis was to verify the possibility that an external force field can be used in the simulation of the protein-folding process, taking into account the diverse nature of the environment that guarantees a structure showing biological activity.

## 1. Introduction

The aquatic environment is a natural environment for proteins and one that conditions biological activity. The vast majority of proteins are water-soluble proteins. Another equally important environment is that of the cell membrane, the characteristics of which (high hydrophobicity) are radically different to those of polar water.

The mechanisms that maintain a cell’s homeostasis in an environment with variable characteristics are critical to the lives of bacteria. The main task is osmoregulation, i.e., the maintenance of a constant level of osmotic pressure. The main goal is to stabilize the electrolyte concentration, leading to fluid balance. An increased transport of water towards environments with higher osmotic pressure can be observed. Such increased transport can result in cell rupture. This process is controlled by proteins called Conductance Mechanosensitive Ion Channels (Msc) [1,2,3,4,5]. Due to their construction, a distinction is made between small conductance mechanosensitive channels (MscS) and large ones (MscL) [6,7,8].

The present work focused on membrane proteins from the group of small conductance mechanosensitive channels (MscS). These proteins enable bacteria to survive under the threat of an osmotic downshock. The presence of the given proteins enables cellular contents to be expelled by opening the pore, preventing cellular rupture. Two molecules, the structures of which were stabilized with a detergent, were analyzed. The aim of the study was to verify the application of the fuzzy oil drop model to assess the structures of individual domains of the extensive structure of the MscS protein.

To assess the structures of the membrane proteins, a modified version of the FOD-M model was used, taking into account the presence of an environment not limited only to the aquatic environment [9]. The FOD model assumes that a polypeptide chain composed of bipolar molecules aims to create a micelle-like structure, concentrating hydrophobic residues in the center with simultaneous exposure of polar residues on the surface [10].

However, obtaining an ideal distribution adapted to the polar environment of water is limited by the lack of freedom of movement of individual amino acids (covalent peptide bonds). Therefore, alignment according to the spherical micelle model is achieved by proteins to different extents. Local deviations from the idealized distribution have been shown to be related to biological activity; a local excess of hydrophobicity is used to build multichain complexes [11], and a local deficit for the selective binding of ligands or substrate [12].

The quantitative measurement of these deviations is determined by using the 3D Gaussian function, which, spread over the protein body, represents an idealized distribution *T*. The *O* distribution observed in the protein, resulting from hydrophobic interactions between amino acids, can be compared with the idealized distribution to reveal the mentioned differences.

The 3D Gaussian (3DG) function represents the maximum concentration of hydrophobicity in the center of the molecule, with values close to zero on the surface (within 3Sigma from the center). Such conditions favor the solubility of the protein [10].

The different environment of the membrane requires the opposite situation in the form of exposure of hydrophobic residues on the surface (contact with the membrane) and, in the case of the channel, free space and the presence of polar residues on the surface of the channel in the central part of the molecule. Therefore, the FOD-M model uses a function of the form 1–3DG to describe such a hydrophobicity distribution. The comparison of the distribution observed in the protein (hydrophobic inter-amino-acid interactions) in the membrane protein with the above-mentioned function can be expressed quantitatively by assessing the status of a given molecule [13].

In membrane proteins, in addition to the domain fully anchored to the membrane, there are outwardly exposed domains (assuming an aqueous environment) that do not exhibit the characteristics of a membrane domain. Therefore, the analysis of such a molecule enables the application of an appropriate model and its verification.

The description of the calculation procedure is given in the Materials and Methods. The work presents a tool for the assessment of the structure of proteins, including membrane proteins in particular. The method may prove useful in research on the properties of membrane proteins, the analysis of which is difficult due to their insolubility.

## 2. Experimental Section

The proteins that were the subjects of the current analysis (Table 1) were selected to represent the structures of membrane proteins serving as channels. The selection of proteins was designated by their different structural and functional forms in order to reveal differences in the interpretation of the obtained results based on the fuzzy oil drop model. Despite performing a similar function, the channels for the transport of various compounds take different structural forms. The list also includes a protein showing the typical system characteristic of water-soluble proteins, with a high match of the hydrophobicity distribution (*O*) to the assumed distribution (*T*) in the FOD model in accordance with 3DG. The presence of this protein was intended to provide an example enabling comparative analysis for the discussed membrane proteins.

The inclusion of MsbA proteins, the structure of which was obtained using peptidiscs, was intended to verify their role as a factor enabling the solubility of membrane proteins. For the purposes of the present analysis, they were used as an example to validate the applicability of the FOD-M model to a wide range of membrane-anchored protein (or membrane-like environment) structures.

The BRSCT protein is a representative of soluble proteins, and therefore opposed to membrane proteins. Its presence was intended to enable comparative analysis for the application of the FOD and FOD-M models.

### 2.1. Description of the FOD Model and Its Modifications FOD-M

This model has been described many times [10]. A brief description is provided here to assist with the interpretation of the results.

The FOD model is an extended version of the oil drop model introduced by Kauzmann [17]. He assumed that the protein is composed of two layers: the outer polar layer and the inner hydrophobic layer. This discrete model was extended to a continuous form by introducing a 3D Gaussian function into the description of the hydrophobicity distribution. This function reflects the high concentration of hydrophobicity in the center of the molecule and the zero level of hydrophobicity at the surface. The values of hydrophobicity decrease from the maximum in the center as we move away from the center. The 3D Gaussian function spread over the body of the protein allows determination of the idealized level of hydrophobicity in the positions of effective atoms (averaged positions of atoms making up the amino acid). This distribution, referred to as *T*, compared with the actual level resulting from inter-amino-acid interactions, referred to as *O*, reveals the degree of similarity and allows for the identification of locations with different characteristics [18]. The *O* distribution expresses dependence on the distance between amino acids and their intrinsic hydrophobicity. Any scale can be applied [10]. After the *T* and *O* distributions are normalized, it is possible to quantify their degree of similarity. This assessment is performed by using the *D_KL_* divergence entropy [19] and introducing a second reference distribution *R*, where each residue is assigned a hydrophobicity level of 1/*n*, where *n* is the number of amino acids in the chain. Such a distribution, contrary to the distribution described by the 3DG function, does not differentiate the levels of hydrophobicity in any way. Therefore, the protein is described with two *D_KL_* values: one for the *O|T* relation and one for the *O|R* relation. Comparing these values allows evaluation of the distances between distributions. A protein for which *D_KL_* (*O|T*) < *D_KL_* (*O|R*) is interpreted as having a hydrophobic concentration in the form of a hydrophobic core. To avoid using two values, the parameter *RD* (Relative Distance), defined as follows, was introduced:

(1) $$RD=\frac{D_{KL}(O|T)}{D_{KL}(O|T)+D_{KL}(O|R)}$$

An *RD* value > 0.5 indicates the presence of generally micelle-like deformation. Identification of positions or segments on the *T* and *O* profiles with divergent courses allows the identification of amino acids and their roles, i.e., the biological function of a given protein.

The hydrophobic environment of the membrane requires a completely different arrangement of the hydrophobicity in a protein in order to be able to permanently interact with the surrounding membrane. Here, the protein should exhibit hydrophobicity on the surface, and, in the case of a channel, polarity in the center (or, due to the free space of the channel, low hydrophobicity). Therefore, to describe the hydrophobicity distribution in a protein domain anchored in the membrane, a function was proposed in the FOD-M model that complements the 3D Gauss function. For calculation purposes, the “inverse” distribution is defined as follows:

*Mi* = *T_MAX_* − *Ti* (2)

where *T_MAX_* is the maximum value in the *T* distribution

This distribution *Mi* should be normalized:

*Mi* = (*T_MAX_* − *Ti*)_*n*_ (3)

where the index *n* denotes the normalization of the distribution.

In fact, the distribution of the membrane-anchored domain is not a simple *Mi* distribution, but a combination of the *T* distribution and the *M* distribution.

Finally, taking into account the possibility of a variable proportion between the distribution *T* and *M*, the outer field is defined as follows:

*Mi* = [*Ti* + K ∗ (*T_MAX_* − *Ti*)_*n*_]_*n*_ (4)

where K is the parameter defining the contribution of the factor expressing the “modification” of the field based on the micelle-like distribution.

All the examples of proteins described later in the results are expressed by the values of the *RD* parameter and the K parameter. The *RD* parameter is interpreted as the degree of matching of the *O* distribution to the *T* distribution in relation to the relative distribution of *R*. It should be noted that this compliance indicates the solubility of the protein in water. On the other hand, the parameter K denotes the degree to which the nonpolar environment (including hydrophobicity in particular) is involved in the generation of a structure with a specific ordering present in the protein.

**Figure 1:**
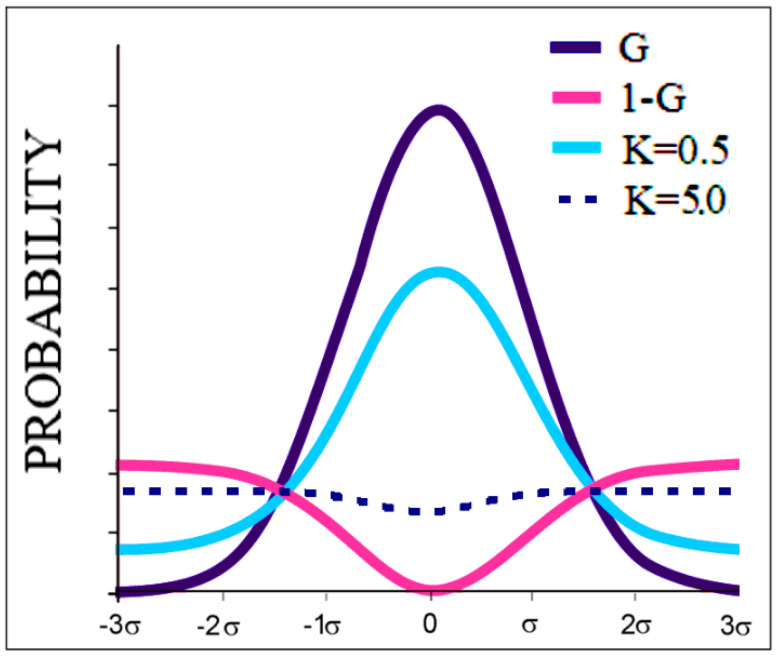
Graphical presentation of the application of the 3DG and 1–3DG functions and the influence of the parameter K = 0.5. The dotted line represents the characteristics of a membrane protein (K = 5.0). The values of K are included only for presentation in a very simplified form. All plots are shown after normalization.

The value of K for a given set of *T* and *O* profiles can be found by searching for the distribution *M* for which *D_KL_* for the relation *O|M* is minimal. The graphic presentation of the model is shown in Figure 1.

It is apparent in light of the research conducted so far that the value of K = 0, meaning the exclusive influence of the aquatic environment, occurs in only a few proteins (including downhill and fast-folding proteins [20]). Most soluble proteins have a K value in the range of 0.2–0.5. In contrast, K values close to 1 are found in membrane proteins that do not act as channels. As is shown in the analysis below, membrane proteins, including those serving as channels, show values of K > 1. This means that the dominant factor in shaping the protein structure is an environment different from the aqueous one, particularly a membrane one.

### 2.2. Tools Used

There are two possible routes of access to the program:

The program allowing calculation of *RD* is accessible upon request on the CodeOcean platform: https://codeocean.com/capsule/3084411/tree (accessed on 15 December 2021). Please contact the corresponding author to get access to your private program instance.

In order to ensure reproducibility of results and provide easy access to the computations discussed in this paper, the authors have also implemented an online tool with which FOD computations can be performed on arbitrary protein structures, including the structures discussed in this paper. The application, implemented in collaboration with the Sano Centre for Computational Medicine (https://sano.science (accessed on 15 December 2021)) and running on resources contributed by ACC Cyfronet AGH (https://www.cyfronet.pl (accessed on 15 December 2021)) in the framework of the PL-Grid Infrastructure (https://plgrid.pl (accessed on 15 December 2021)), provides a web wrapper for the abovementioned computational component and is freely available at https://hphob.sano.science (accessed on 15 December 2021).

**Figure 2:**
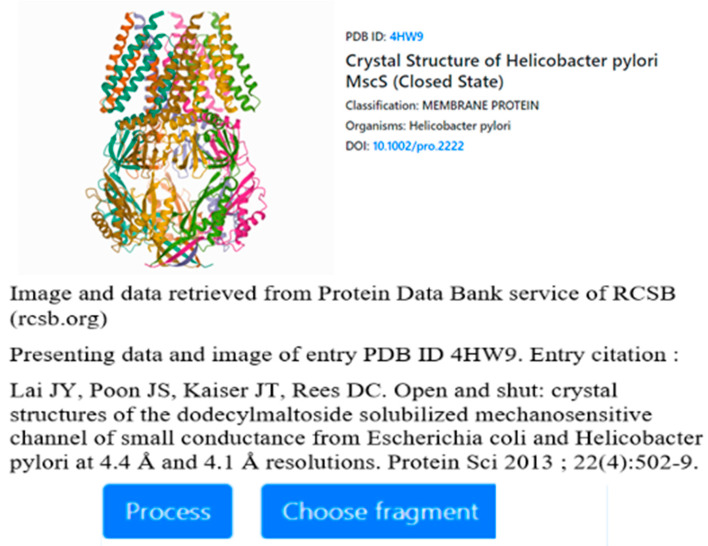
User interface of the HPHOB online tool which provides the ability to compute FOD model parameters for arbitrary protein structures. The tool is available at https://hphob.sano.science (accessed on 15 December 2021).

The tool enables users to select a protein structure by entering its PDB identifier, to select specific parts of the protein (including individual chains and secondary folds, all the way down to individual residues), and finally to run the FOD computation on the selected fragments in order to obtain *RD* and hydrophobicity distribution data (Figure 2).

## 3. Results

### 3.1. Analysis of Exemplary MscS Proteins

The characteristics of the HpMscS and EcMscS proteins are presented in Table 2, wherein the values of the *RD* and K parameters are given.

**Figure 3:**
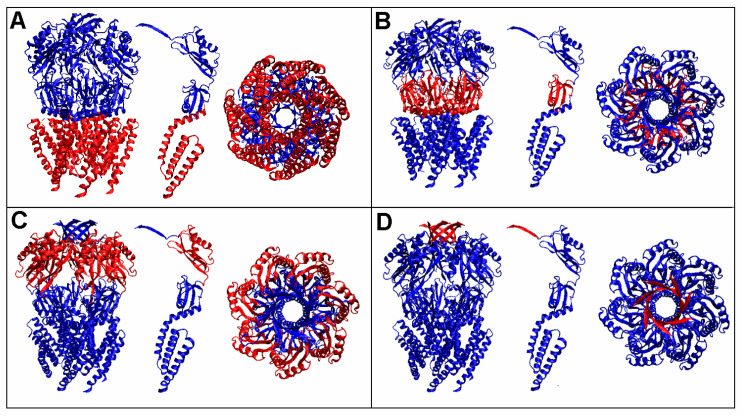
The arrangement of domains in HpMscS (4HW9) and their identification used in the present work. Two perspectives and an appropriately oriented structure of a single chain in the central part are shown. (**A**) Domain 1, defined as DD1 if analyzed as a set of seven fragments of the chain and D1 if analyzed as an independent structural unit in a single chain (central position in each of the windows). (**B**) Domain 2, defined as DD2 if analyzed as a set of seven fragments of the chain and D2 if analyzed as an independent structural unit in a single chain. (**C**) Domain 3, defined as DD3 if analyzed as a set of seven fragments of the chain and D3 if analyzed as an independent structural unit in a single chain. (**D**) Domain 4, defined as DD4 if analyzed as a set of seven fragments of the chain and D4 if analyzed as an independent structural unit in a single chain.

As the characteristics of the two analyzed MscS proteins were similar, only HpMscS (4HW9) is discussed in detail in the following sections. In order to facilitate the identification of its individual components, appropriate nomenclature is proposed in Figure 3, where the system to identify domains is presented.

The designation DD# identifies a set of corresponding domains as part of a complex, while the designation D# expresses the status of a domain treated as an individual structural unit. In the case of DD#, the Gaussian function was defined for a set of seven relevant domains. In the case of D#, the Gaussian function was defined for an individual structural unit. The others indicate the status of the fragment as a component mentioned in the header.

The values of the *RD* and K parameters given in the section entitled “COMPLEX” express the statuses of the complexes formed by the set of all chains and the seven domains, respectively. A 3D Gaussian function (DD# designation) was generated for the whole complex and for the complexes produced by a given set of domains.

The status of the domain set and the chain as part of the entire complex is also given (in the part of the table entitled “Fragments in complex”). The given values define the contributions and roles of individual fragments as components of the complex structure. A separate Gaussian function was not generated to determine this status. This status was determined after comparative analysis of the fragments of the *T* and *O* distributions for the selected part of the complex.

Similarly, in the section entitled “Chain individual”, the status of a single chain (3D Gauss generated for the chain) is given, while the values given in this part of the table define the statuses of individual domains as members of a single chain.

The section “Domains individual” gives the statuses of domains treated as individual structural units. A 3D Gaussian function was generated for each of them. Domains treated in this way are referred to as D#.

**Figure 4:**
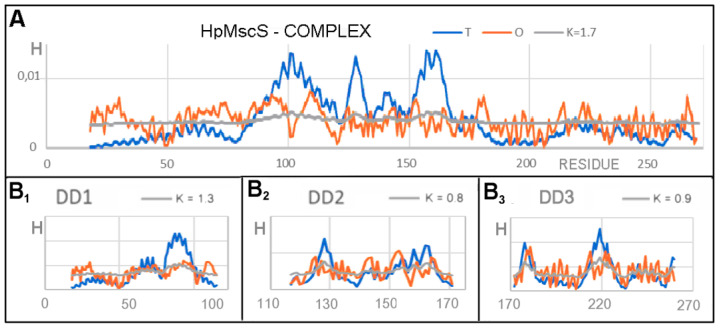
Set of *T*, *O*, and *M* profiles for determining the status of the A chain and domains in the complex, the 3DG function, and its modifications generated for the complex. (**A**) The status of the complex represented by chain A. The complete *T*, *O*, and *M* profiles for the complex are the profiles from (**A**) replicated seven times. For clarity of the graphics, only chain A is shown. (**B**) Set of domains: (**B_1_**) DD1, (**B_2_**) DD2, (**B_3_**) DD3; here also, a fragment duplicated seven times is shown rather than presenting the entire complex.

Analysis of the *T*, *O*, and *M* profiles for the complex (represented by the A chain to avoid duplication of the same profile seven times) showed elevated levels in the N-terminal segment DD1 domain (Figure 4A). The presence of a channel in the form of a deficit level of hydrophobicity in the locations of expected maximum hydrophobicity concentration was clearly marked. The C-terminal segment showed a relative alignment of the levels of *T* and *O*. This is the region of the domains beyond the membrane. A high value of K > 1 implies the need for a significant modification of the target distribution, a distribution that expresses the characteristics of an external field that differs from the idealized field as defined for globular proteins. A significant excess of hydrophobicity in the N-terminal section (DD1 domain region) suggested the participation of a hydrophobic environment in the generation of the structural form. The central part showed a definite hydrophobicity deficit, indicating the presence of large-sized canal chambers. The C-terminal segment (about 1/3 of the chain length) showed a relative agreement of the *T* and *O* distributions.

The set of profiles calculated for the domain defined as DD1 (Figure 4(B_1_)) (set of seven chain fragments) showed excess hydrophobicity on the N-terminal fragment itself, but also precisely determined the presence of a channel in the segment with a significantly underestimated hydrophobicity (C-terminal fragment of this domain). In relation to the status of the complex, a decrease in the value of K was observed.

The characteristics of the DD2 domain (Figure 4(B_2_)) showed the presence of the channel, although it was definitely more clearly visible on the profiles shown in Figure 4A. The DD3 domain seemed to represent the status with the distribution *O* closest to the expected *T* distribution (Figure 4(B_3_)), although the presence of the channel in the form of a local hydrophobicity deficit was visible.

The DD2 and DD3 domains showed reduced K values which were relatively high compared to the statuses of the soluble proteins. This was mainly due to the presence of a channel expressed as a significant local deficit in hydrophobicity.

**Figure 5:**
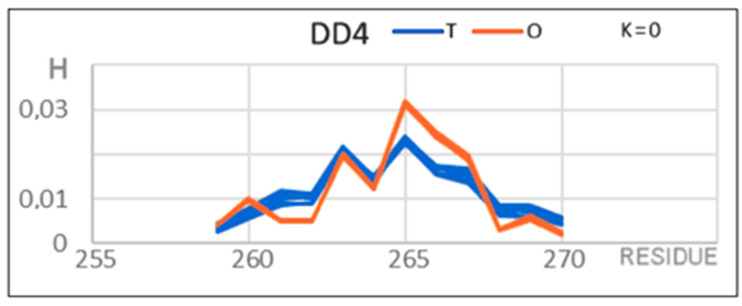
Set of *T* and *O* profiles for the DD4 domain in HpMscS. There is a clear high agreement of the idealized (*T*) and observed (*O*) distribution. The determined value of K = 0 does not require the determination of the *M* distribution.

The DD4 domain deserves special attention (Figure 5). It consisted of a set of seven short segments with a beta structure forming a typical beta-barrel (Figure 3D). From the point of view of the FOD model, it represents a distribution typical of water-soluble globular proteins, which is indicated by the high compatibility of the *T* and *O* distributions expressed by the low value of *RD* and K = 0.

In summarizing the characteristics of the complex and components in the form of complexes composed of domains, it should be noted that from the point of view of the complex, the characteristics of the set of *T* and *O* profiles showed a specific system in which the identification of the membrane domain was clear and the presence of the channel was also unambiguous.

The status of the domain set (DD) seemed to be more mutually ordered, where, for example, the excess of hydrophobicity observed in the *T*, *O*, and *M* profiles (Figure 4A) was consumed in the case of the DD1 domain complex on interchain interactions, representing the excess hydrophobicity in the N-terminal and C-terminal sections.

**Figure 6:**
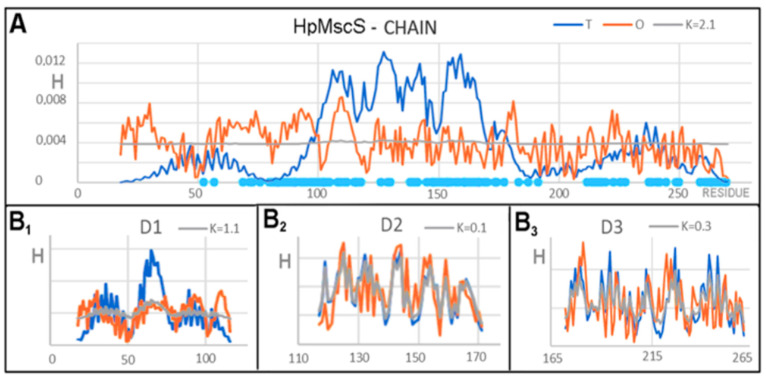
A set of *T*, *O*, and *M* profiles for (**A**) the A chain treated as an individual building unit. The light blue positions on the x axis distinguish the positions of the interchain interactions involved in the final structure of the complex; (**B**) a set of profiles for domains treated as individual structural units: (**B_1_**)—D1, (**B_2_**)—D2, (**B_3_**)—D3.

Characteristics of the structure of a single chain indicated a folding significantly deviating from the globular system with a clear exposure of hydrophobicity in the N-terminal segment and a substantial deficit in the middle segment, with a relatively matched *O* distribution in the C-terminal segment (Figure 6A). Such a distribution with a very high value of K = 2.1 means that this structure could not be achieved in an aquatic environment. The high value of K suggests a significant share of environmental factors with changed characteristics in relation to the aquatic environment.

The part of Table 2 referred to as “Fragments in complex” gives the characteristics of the fragments mentioned, which constituted components of the entire structure of the complex. In other words, the parameters presented herein determine the local roles played by the given fragments. The values of *RD* and K were obtained by normalizing selected fragments from the profiles obtained for the complex. The statuses of these fragments appeared to be comparable to that of the set of domains (denoted as DD) with the exception of DD4, which occupied a superficial localization and, as shown by the profile set (Figure 4A), low hydrophobicity was expected.

The next analysis was the status of a single chain (first line of the “Individual chain” part of Table 2). The values of the *RD* and K parameters indicate that the chain structure was far from the statuses represented by globular proteins. The stretched form with only locally marked higher packings was in no way close to the micelle-like form that is expected for a chain folding in an aqueous environment. The high incompatibility of the *O* distribution with the *T* distribution was due to a significant excess of hydrophobicity in the N-terminal part. There was a clear deficit of the expected high concentration of hydrophobicity in the central part of the chain. A relatively similar distribution of *T* to the distribution of *O* was observed in the C-terminal part (Figure 6A). Local high levels of excess hydrophobicity are likely partly used for interchain interaction in both the N- and C-terminal fragments. The central, section showing a significant deficit in hydrophobicity, probably consumed the hydrophobic residues present there in part for interchain interaction.

The value of K = 2.1, which determined the status of the discussed chain, suggests complete independence from the aquatic environment. The *M* distribution, which almost took the form of the *R* distribution, also drew attention. This situation is discussed later in this work.

The analysis of the statuses of individual domains treated as individual structural units suggested the course of the chain-folding process. The domains D2 and D3 (Figure 6(B_2_,B_3_)) indicated that these domains generate a micelle-like system with a relatively high consistency of the *T* and *O* distributions at low K values. This means that these domains can form spontaneously in the aquatic environment by striving to create a local hydrophobic nucleus with a polar surface (micelle-like system) (Figure 6(B_2_,B_3_)).

The statuses of individual chains and the domains present in them, treated as components of the entire structure, clearly differentiated the characteristics of subsequent domains. The status of the chain as a component of the complex was comparable to that of the entire complex. Among the domains, the status of the membrane domain was clearly different. Here, both the values of *RD* and K were clearly high, while in the other domains the values were much lower (Figure 6(B_1_)). This means that for the individual sections of the chain that make up the domains, there was a much better match to the *T* distribution characteristic of the aquatic environment. Attention was drawn to the C-terminal fragment, with its very low values of *RD* and K. The status of this segment constructed by seven short C-terminal fragments represents the status expressed by K = 0.0.

The analysis of the statuses of individual domains treated as individual structural units very clearly differentiated the N-terminal domain, i.e., the membrane domain. The remaining domains showed a status characteristic of the aquatic environment, showing the presence of a hydrophobic nucleus and the exposure of polar residues on the surface. Some of these were engaged to interact with analogous fragments of adjacent chains. It appears that a local excess of hydrophobicity in the individual D2 and D3 domains, e.g., positions 149–151, 215–222, and 234–236, is used for the purpose of complexing the adjacent chain, thus starting to form a larger complex. These sections in the structure of the complex fit into a consistent order (sections 149–151 and 234–236), while the local excess (section 215–222) noted in the structure of a single D3 domain in the structure of the DD3 domain showed a hydrophobicity deficit, probably constituting a channel wall within the DD3 domain (Figure 4(B_3_) and Figure 6(B_3_)).

The dissimilarity of the membrane domain in the form of both the DD1 and D1 complexes results from a marked excess of hydrophobicity over the entire section of this domain. This was present both in the form of a complex and in the single domain. Very high K values indicated a significant share of the environment and environment modifying factor for this domain. This suggests the need for the direct presence of a membrane to direct the shaping process towards the expected direction for the membrane-anchored domain. This statement is self-evident. However, it expresses the correctness of the model used (Equation (4)).

Table 2 lists two membrane proteins with similar biological functions. The authors defines the status of the HpMscS (4HW9) protein as closed and EcMscS (4HWA) as open. Table 2 shows the locations of the differences between the statuses of these two forms, especially those observed in the structure of a single chain. However, this observation cannot be interpreted in the context of biological function due to the low degree of sequence identity (33%) [14]. Nevertheless, the structural analysis justifies the use of the FOD and FOD-M models to describe the structures from the MscS group.

### 3.2. Representative of Proteins from the MsbA Group

The structure of the transmembrane protein discussed here, called translocase (Lipid A export ATP-binding/permease protein MsbA), is the result of research on the adjustment of experimental conditions to enable soluble forms of membrane proteins to be obtained. The discussed structures were obtained in the environment of the β-dodecylmaltoside detergent, mimicking the membrane environment [15].

The analysis expressed by the parameters of the FOD and FOD-M models creates comparative possibilities for difficult-to-obtain experimental materials for research on membrane proteins.

The structures of the discussed proteins are homodimers, consisting only of the membrane domains described by the parameter set (Table 3).

Very high values of both *RD* and K resulted from the fact that the structure was completely subordinated to the conditions of the membrane. Both the complexes and single chains and domains present in them showed significant divergences from the distributions expected for the structures characteristic of soluble proteins (Table 3).

From the point of view of the analysis based on the FOD and FOD-M models, both proposed structures showed a high degree of similarity to each other, differing slightly in terms of the parameter values themselves. However, these differences did not cause any discrepancy in interpretation.

**Figure 7:**
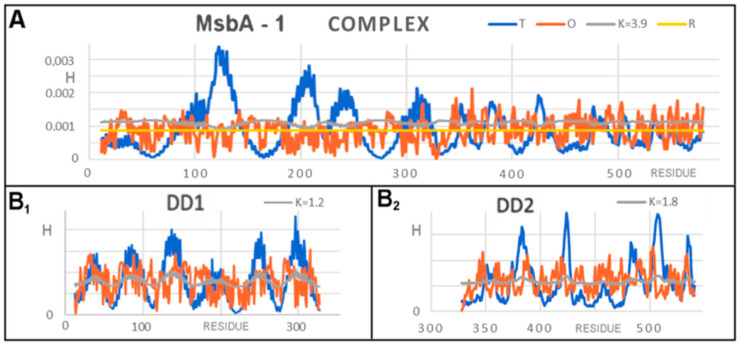
A set of profiles for the 6UZL homodimer 3DG and 1–3DG function assigned to the complex. (**A**) Distribution in chain A as a representative of the complex. Additionally, distribution *R* is marked. (**B**) Distributions in the domains: (**B_1_**) DD1, (**B_2_**) DD2.

For the analyses based on FOD and FOD-M, the example model presented is a very interesting example for the interpretation of the K parameter value. As mentioned before, a K > 1 value is expected for membrane proteins. Values of K > 3 suggest a very high share of the membrane-like factor in shaping the structures of these proteins (Figure 7).

The discussed example (in particular 6UZL) introduced a new observation resulting from the value of K > 3. It should be noted that the *R* distribution (without any differentiation) for this protein was obtained for K = 2.8. This means that the distribution for this K value represents a system with a uniform hydrophobicity distribution (Figure 7A).

In the set of *T*, *O*, and *M* profiles, attention was drawn to the sections where, instead of maxima in the *T* distribution, minima appeared in the *M* distribution. Mathematically, this was the result of very high K values (K > 3). In the case of this membrane protein (dimer), in the center, where the hydrophobicity maximum was expected according to the FOD model, a local minimum appeared. This means that the model of the “inverse” Gaussian function applied here. All sections on the *M* profile (Figure 7A) are distinguished in the figures shown: ice blue for D1 and red for D2. The *R* distribution is also given in Figure 7A. The *R* distribution represents the state where the effect of the 3DG function balances the effect of the TMAX-Ti (1–3DG) function. This state was obtained for K = 2.8. The optimal value of K was much larger for the system. This demonstrates the advantage of the “inverse” Gaussian function. Interpretation of this observation suggested that polar residues are present in the central part. There are also hydrophobic residues which, due to the proximity of the free space of the channel, effectively exhibited a much lower level of hydrophobicity. As a result, an area (chain segment) was obtained with a clear hydrophobicity deficit, indicating the location of the channel. Comparative analysis with an idealized distribution consistent with 3DG, as observed in the case of globular soluble proteins, in the discussed protein (dimer) gives an accurate picture of the situation of the membrane protein (hydrophobicity exposure) in the presence of a channel in the central part (polar residues) (Figure 7(B_1_,B_2_)).

**Figure 8:**
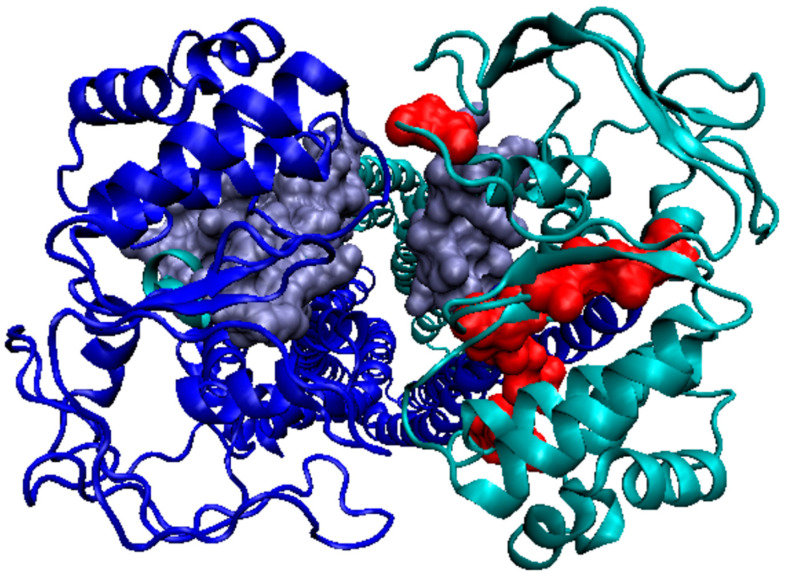
3D structure of a homodimer (6UZL). In chain A (turquoise), sections showing local minima in places expected to be local maxima were distinguished. Chain A: turquoise, ice blue: sections within DD1 domains, red: sections within DD2 domains.

The assessment of the distributions in individual domains did not show this effect to such a strong extent, suggesting that the channel clearly appears only as a result of joining two chains (Figure 8).

**Figure 9:**
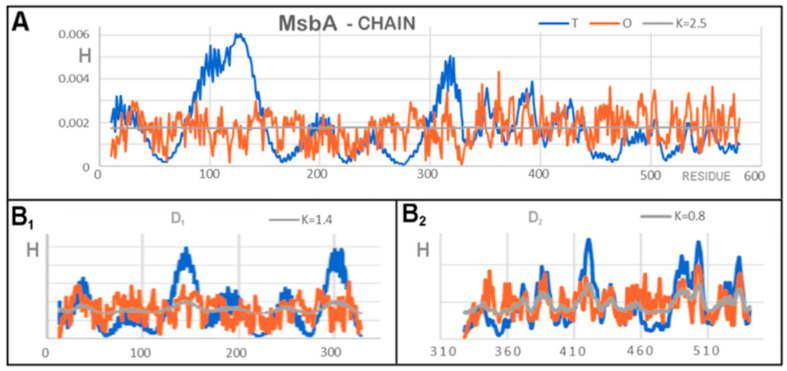
A set of *T*, *O*, and *M* profiles for 6UZL for a single chain and domains treated as individual structural units. (**A**) Chain A. (**B**) Domains (**B_1_**) D1, (**B_2_**) D2.

The characteristics of the *T*, *O*, and *M* distributions for a single chain revealed a significant excess of hydrophobicity along the entire length of the chain, thus expressing a structural system that was far from globular. There were clearly deficiencies in hydrophobicity in the area of the ultimate location of the channel. The value of K = 2.5 was characteristic for the distribution of *T*, *O*, and *M* for a single chain, which in this case meant that the distribution *M* (optimal as a target for folding this chain) coincided with the distribution *R* (Figure 9A). The distribution *M* took the form of a straight line. This represents folding in an environment treated as a kind of “vacuum”, i.e., no external factors had any influence on the formation of this chain, generating neither a hydrophobic nucleus nor its inverse. Profiles (Figure 9(B_1_,B_2_)) revealed different degrees of accordance between the *T* and *O* distribution measured by K, which were higher for the membrane domain (D1) and lower for the external domain (Figure 9(B_2_)).

Due to the K > 3 value for the complex and the *R* distribution obtained for appropriate modification of the *T* distribution, the protein discussed here is a valuable subject for FOD-based analysis and FOD-M modeling.

### 3.3. Protein with an O Distribution Consistent with the T Distribution

The last example discussed here is a DNA-binding protein called rap1, which is a domain of the BRC (Breast Cancer) protein (PDB ID 2L42 [16]). This protein was included in the present analysis as an example of a structure representing a highly compatible *O* distribution versus a *T* distribution with a low K value, and thus as an example different from those previously discussed. This was to allow (at least narrowly) comparative analysis with an example of a soluble protein with a hydrophobic nucleus and a polar surface.

The second reason this protein is interesting is that it is identified as having a disordered protein status along its entire chain length (97 aa). The status of this single-chain protein in terms of the presence of the disordered form is discussed in the DisProt database [21,22].

In contrast, the FOD-based analysis model evaluated this protein as highly ordered from the point of view of the structure of the hydrophobic nucleus.

The parameters *RD* = 0.387 for the T–O–R relationship and a very low value of K = 0.2 suggested a high order of hydrophobicity in line with the micelle-like form, i.e., a typical arrangement characteristic of proteins that are soluble and fold under the influence and active participation of the aquatic environment.

The classification in the disordered protein category was due to a very low secondary structure content (only 29% of the chain length forms a secondary structure with a chain length of 97 aa). The absence of disulfide bonds deprives the protein of the stabilization resulting from the presence of this type of covalent bond. The dominant source of stabilization is therefore the presence of a hydrophobic nucleus. Changes in the external environment, perhaps even small ones, could be a destabilizing factor for this protein. This probably explains the observed structural instability of this protein and its presence in the DisProt base.

**Figure 10:**
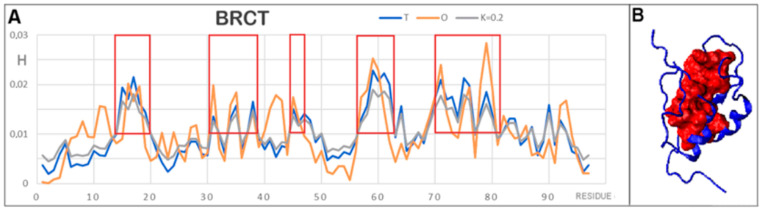
Characteristics of BRCT. (**A**) The *T*, *O*, and *M* profiles for BRCT show a high agreement of the distributions. The boxes distinguish the items constituting the components of the hydrophobic nucleus. These were determined using the criterion of high values of *Ti* and *Oi*. The frames distinguish sections that are part of the hydrophobic nucleus. (**B**) 3D structures of the BRCT protein (PDB ID 2L42) with the highlighted hydrophobic nucleus (red) according to the identification shown in (**A**).

In Figure 10A, the criterion of *Ti* and *Oi* above 0.01 was used as the compliance criterion for a high level of hydrophobicity (framex in Figure 10A). Thus, the composition of the hydrophobic nucleus shown in Figure 10B as a red form (space-filling) was identified. The navy blue fragments in this figure represent surface and intermediate-level residues. Thus, the micelle-like form present in the structure of the protein in question is made visible.

## 4. Discussion

The influence of the environment on protein folding is a critical factor in obtaining a structure with a specific biological activity. The model used here, based on the similarity of the folding process to the micellization process, results from the nature of amino acids as bipolar molecules that seek to generate a micelle-like structure. The degree to which such a form is obtained depends on the amino acid sequence, which in some cases precludes the generation of such a micelle-reproducing structure. Hence, local maladjustments which are difficult to predict appear to play significant roles in biological activity; in consequence, these maladjustments appear to be highly specific. A part of the protein body accordant with micelle-like construction must be present to ensure a protein’s solubility (for water-soluble proteins). These parts appear similar in many proteins, constructing the protein surface (polar residues). Specific unique forms of maladjustment are varied in numerous proteins. Thus is the code for biological activity.

The phrase “sequence determines the structure of a protein” may be replaced with “sequence determines the form of maladjustment to a spherical micelle”. Restoration of the micelle-like structure would result in the disappearance of any possibility of interaction (except for random polar and charge interactions). The type and degree of mismatch of the *O* distribution with the *T* distribution is of critical importance in determining the specificity of a given protein. This specificity also includes the susceptibility to the influence of the environment, including the participation of the nonpolar environment, in particular the hydrophobic environment of the membrane.

The application of the FOD and FOD-M models proposed here is not only aimed at assessing the status of a given molecule (complex), but also constitutes a proposal for a definition and mathematical record of the external field expressing a protein’s environment. Including these models in the process of simulating the polypeptide chain folding process will allow not only for protein structures to be correctly predicted, but also the question of why proteins fold the way they do to be answered The treatment of the *M* distribution as a “target” or “matrix” type factor in achieving the goal of appropriate hydrophobicity ordering (including disorder) in computer simulations of the protein-folding process should be helpful in the appropriate specific orientation of this process.

It is recommended to simulate the folding process in the presence of an external field with a variable K parameter in order to adjust the hydrophobicity distribution for various external conditions. The postulated method of multiple criteria optimization [23], taking into account nonbinding interactions as one function subjected to optimization, and the second function expressing the matching of the order within the molecule according to the influence of the environment (for variable K values), seems to be a justified solution. The analysis presented here seems to support such an opinion.

The multiple criteria optimization postulated in [23] taking into account two functions, (1) nonbinding interactions and (2) active participation of the environment, seems to be the justified solution. This type of optimization leads to a solution expressing consensus between these two factors. A few simulations should be performed for different K values to take into account different external conditions.

It is also expected that the method presented here can be used by researchers of the biological activity of membrane proteins (and other proteins operating under conditions other than polar water).

The analysis presented here was made possible thanks to the evolving techniques of structure identification, which is difficult due to the nonaqueous environment excluding many experimental techniques [24,25,26,27,28]. Analyses are developed towards both the detailing [29] and generalization of protein structure [30]. Each available structure in PDB enables an analysis, such as the one presented in the present work, due to the necessary knowledge of the spatial position of each (heavy) atom [31].

Structural analyses are carried out from the point of view of the specificity of helical systems characteristic of membrane proteins [32,33,34]. The object of the analysis is also the significant content of the membrane, without which the membrane protein loses its specificity; hence the analyses focused on the participation of detergents, membrane-mimicking factors, and other specifics ensuring the construction of membrane proteins to ensure their biological function [35,36]. The issues related to the identification of functional elements of the construction of complex structures, as well as the study of the influence of the membrane protein environment through introduction of external factors other than the classic membrane, remain closely related to the model presented here [30,35,36]

## 5. Conclusions

The method proposed and used herein to assess the status of membrane proteins, based on the structure of the external field representing the conditions resulting from the specificity of the environment and external conditions, seems to be potentially widely applicable. Other proteins analyzed using the FOD model and its FOD-M modification justify this statement [9,13]. The proposed form of the external field can be used to describe and analyze the structure of any protein. It also proposes a form of mathematical notation of the specificity of the environment that actively influences the final form of the folding protein structure.

The aim of the paper is to reveal the possibility to interpret the results based on fuzzy oil drop model particularly of its modified version (FOD-M). Application to many different proteins and their different biological functions suggests the universal character of the presented model. As it is shown in this paper—the differentiation of the specificity of membrane and cytoplasmic domains—clearly identified by FOD-M model is a good example to prove this suggestion. Application of FOD-M model to amyloids allows differentiation of two scenarios of amyloid transformation [37,38].

## Figures and Tables

**Table 1:** List of proteins included in the analysis. MD: membrane domain, PMD: outside-membrane domain, 1D: single-domain structure.

Protein Code PDB	PDB ID	Organism	Characteristics	Ref.
HpMscS	4HW9	*Helicobacter Pylori*	MD, PMD	[14]
EcMscS	4HWA	*E coli*	MD, PMD	[14]
MsbA-1	6UZ2	*E coli*	MD	[15]
MsbA-2	6UZL	*E coli*	MD	[15]
BRCT domain	2L42	*Saccharomyces cerevisiae*	1D Water-soluble	[16]

**Table 2:** A set of parameter values describing the statuses of the complexes, chains, and domains of proteins representing the mechanosensitive channels of HpMscS and EcMscS, treated as components of the complex and as individual structural units.

HpMscS (4HW9)-CLOSED			EcMscS (4HWA)-OPEN		
Fragment	*RD*	K	K	*RD*	Fragment
COMPLEX					
Complex	0.766	1.6	1.5	0.770	Complex
DD1 18–116	0.782	1.2	1.0	0.763	DD1 25–126
DD2 117–171	0.625	0.8	0.9	0.680	DD2 127–179
DD3 172–260	0.627	0.9	1.0	0.642	DD3 180–270
DD4 261–272	0.255	0.0	0.0	0.090	DD4 271–280
Fragments in complex					
18–272	0.781	1.7	1.7	0.791	25–280
18–116	0.843	1.6	1.8	0.873	25–126
117–171	0.613	0.6	0.4	0.572	127–179
172–260	0.600	0.6	0.8	0.635	180–270
261–272	0.508	0.4	0.3	0.493	271–280
Chain individual					
18–272	0.846	2.1	1.4	0.791	25–280
18–116	0.902	2.6	2.2 R	0.897	25–126
117–171	0.738	2.1	0.2	0.560	127–179
172–260	0.639	1.0	1.4	0.629	180–270
261–267	0.738	2.1	1.0	0.801	271–280
Domains individual					
D1 18–116	0.725	1.1	0.9	0.690	25–126
D2 117–171	0.339	0.1	0.1	0.344	127–179
D3 172–260	0.472	0.3	0.3	0.452	180–270
D4 261–267	0.430	0.1	0.1	0.135	271–280

**Table 3:** A set of parameters that define the MsbA status for forms obtained in the environment of β-dodecylmaltoside, mimicking the membrane environment. D# means another domain treated as an individual structural unit; DD# means the status of a set of domains in a homodimer.

FRAGMENT	6UZ2		6UZL	
	*RD*	K	K	*RD*
COMPLEX				
CHAINS A + B	0.822	3.9	3.4	0.823
DD1	0.717	1.2	1.2	0.724
DD2	0.782	1.8	1.8	0.774
INDIVIDUAL UNITS				
Chain A	0.808	2.4	2.5	0.812
D1	0.771	1.4	1.4	0.771
D2	0.687	0.9	0.8	0.659

## Data Availability

All data can be available on request addressed to corresponding author. The program allowing calculation of *RD* is accessible on GitHub platform: https://github.com/KatarzynaStapor/FODmodel and on https://hphob.sano.science (accessed on 15 December 2021).

## References

[1] Hannah R., Malcolm H.R., Paul B.P., Joshua A., Maurer J.A. (2015). The mechanosensitive channel of small conductance (MscS) functions as a Jack-In-The Box. Biochim. Biophys. Acta (BBA)-Biomembr..

[2] Peyronnet R., Tran D., Girault T., Frachisse J.-M. (2014). Mechanosensitive channels: Feeling tension in a world under pressure. Front. Plant Sci..

[3] Guharay F., Sachs F. (1984). Stretch-activated single ion channel currents in tissue-cultured embryonic chick skeletal muscle. J. Physiol..

[4] Wilson M.E., Maksaev G., Haswell E.S. (2013). MscS-like mechanosensitive channels in plants and microbes. Biochemistry.

[5] Corry B., Martinac B. (2008). Bacterial mechanosensitive channels: Experiment and theory. Biochim. Biophys. Acta (BBA)-Biomembr..

[6] Gullingsrud J., Schulten K. (2004). Lipid bilayer pressure profiles and mechanosensitive channel gating. Biophys. J..

[7] Gullingsrud J., Schulten K. (2003). Gating of MscL studied by steered molecular dynamics. Biophys. J..

[8] Gullingsrud J., Kosztin D., Schulten K. (2001). Structural determinants of MscL gating studied by molecular dynamics simulations. Biophys. J..

[9] Roterman I., Stapor K., Fabian P., Konieczny L. (2021). The Functional Significance of Hydrophobic Residue Distribution in Bacterial Beta-Barrel Transmembrane Proteins. Membranes.

[10] Kalinowska B., Banach M., Konieczny L., Roterman I. (2015). Application of Divergence Entropy to Characterize the Structure of the Hydrophobic Core in DNA Interacting Proteins. Entropy.

[11] Dygut J., Kalinowska B., Banach M., Piwowar M., Konieczny L., Roterman I. (2016). Structural Interface Forms and Their Involvement in Stabilization of Multidomain Proteins or Protein Complexes. Int. J. Mol. Sci..

[12] Banach M., Konieczny L., Roterman I., Irena R.-K. (2020). Ligand binding cavity encoded as a local hydrophobicity deficiency. From Globular Proteins to Amyloids.

[13] Roterman I., Stapor K., Fabian P., Konieczny L., Banach M. (2021). Model of Environmental Membrane Field for Transmembrane Proteins. Int. J. Mol. Sci..

[14] Lai J.Y., Poon Y.S., Kaiser J.T., Rees D.C. (2013). Open and shut: Crystal structures of the dodecylmaltoside solubilized mechanosensitive channel of small conductance from Escherichia coli and Helicobacter pylori at 4.4 Å and 4.1 Å resolutions. Protein Sci..

[15] Angiulli G., Dhupar H.S., Suzuki H., Wason I.S., Van Hoa F.D., Thomas W.T. (2020). New approach for membrane protein reconstitution into peptidiscs and basis for their adaptability to different proteins. Elfie.

[16] Zhang W., Zhang J., Zhang X., Xu C., Tu X. (2011). Solution structure of Rap1 BRCT domain from Saccharomyces cerevisiae reveals a novel fold. Biochem. Biophys. Res. Commun..

[17] Kauzmann W. (1959). Some factors in the intepretation of protein denaturation. Adv. Prot Chem..

[18] Levitt M. (1976). A simplified representation of protein conformations for rapid simulation of protein folding. J. Mol. Biol..

[19] Kullback S., Leibler R.A. (1951). On information and sufficiency. Ann. Math. Stat..

[20] Banach M., Stapor K., Konieczny L., Fabian P., Roterman I. (2020). Downhill, Ultrafast and Fast Folding Proteins Revised. Int. J. Mol. Sci..

[21] Hatos A., Hajdu-Soltész B., Monzon A.M., Palopoli N., Lucía Á.L., Aykac-Fas B., Bassot C., Benítez G.I., Bevilacqua M., Chasapi A. (2020). DisProt: Intrinsic protein disorder annotation in 2020. Nucleic Acids Res..

[22] www.disprot.org.

[23] Konieczny L., Roterman I., Irena R.-K. (2012). Conclusion. Protein Folding In Silico.

[24] Hong M., Zhang Y., Hu F. (2012). Membrane protein structure and dynamics from NMR spectroscopy. Annu Rev. Phys. Chem..

[25] Das B.B., Park S.H., Opella S.J. (2015). Membrane protein structure from rotational diffusion. Biochim. Biophys. Acta.

[26] Moraes I., Evans G., Sanchez-Weatherby J., Newstead S., Stewart P.D. (2014). Membrane protein structure determination—The next generation. Biochim. Biophys. Acta.

[27] Pedro A.Q., Queiroz J.A., Passarinha L.A. (2019). Smoothing membrane protein structure determination by initial upstream stage improvements. Appl. Microbiol. Biotechnol..

[28] Pandey A., Shin K., Patterson R.E., Liu X.Q., Rainey J.K. (2016). Current strategies for protein production and purification enabling membrane protein structural biology. Biochem. Cell Biol..

[29] Hendrickson W.A. (2016). Atomic-level analysis of membrane-protein structure. Nat. Struct Mol. Biol..

[30] Overduin M., Esmaili M. (2019). Memtein: The fundamental unit of membrane-protein structure and function. Chem. Phys. Lipids.

[31] Tusnády G.E., Dosztányi Z., Simon I. (2004). Transmembrane proteins in the Protein Data Bank: Identification and classification. Bioinformatics.

[32] Fuchs A., Frishman D. (2010). Structural comparison and classification of alpha-helical transmembrane domains based on helix interaction patterns. Proteins.

[33] Neumann S., Fuchs A., Mulkidjanian A., Frishman D. (2010). Current status of membrane protein structure classification. Proteins.

[34] Shimizu K., Cao W., Saad G., Shoji M., Terada T. (2018). Comparative analysis of membrane protein structure databases. Biochim. Biophys. Acta (BBA)-Biomembr..

[35] Choy B.C., Cater R.J., Mancia F., Pryor E.E. (2021). A 10-year meta-analysis of membrane protein structural biology: Detergents, membrane mimetics, and structure determination techniques. Biochim. Biophys. Acta (BBA)-Biomembr..

[36] Roel-Touris J., Jiménez-García B., Bonvin A.M.J.J. (2020). Integrative modeling of membrane-associated protein assemblies. Nat. Commun..

[37] Roterman I., Stapor K., Fabian P., Konieczny L. (2021). In Silico Modeling of the Influence of Environment on Amyloid Folding Using FOD-M Model. Int. J. Mol. Sci..

[38] Roterman I., Stapor K., Gądek K., Gubała T., Nowakowski P., Fabian P., Konieczny L. (2021). On the Dependence of Prion and Amyloid Structure on the Folding Environment. Int. J. Mol. Sci..

